# High-resolution in-beam video rate imaging with the ClearXCam for synchrotron beam­lines

**DOI:** 10.1107/S1600577526001438

**Published:** 2026-03-24

**Authors:** Tyler M. Forgacs, Jesse Brown, Erik M. Muller, Erik R. Farquhar, Manpuneet Kaur Benipal, Anna Zaniewski

**Affiliations:** a Advent Diamond, Inc., 7700 South River Parkway, Tempe, AZ85284, USA; b Brookhaven National Laboratory Upton NY11973-5000 USA; c Center for Synchrotron Biosciences Case Western Reserve University School of Medicine, 10900 Euclid Avenue Cleveland OH44106 USA; RIKEN SPring-8 Center, Japan

**Keywords:** synchrotron instrumentation, diamond sensor, beam-positioning monitor, beam profile monitor, imaging in beam monitor

## Abstract

The ClearXCam detector is a new video rate imaging in-beam monitor based on a diamond sensor. Imaging with 2304 effective pixels is achieved by sequentially biasing one of 48 metal stripes on one side of a diamond sensor, while reading out the current from 48 stripes on the other side of the sensor. This system was characterized for real-time X-ray beam diagnostics at synchrotron beam­line 17-BM at the National Synchrotron Light Source II. Significant results include: detection of in-beam structure *via* the imaging mode, beam focusing in one minute with real-time imaging feedback during a focusing event, linearity over five orders of magnitude, validation of a fast mode operating at 100 Hz, and sub-micron beam-positioning resolution. The system is now available commercially.

## Abstract 2

The ClearXCam is a diamond-based camera for synchrotron X-ray beams. This imaging detector has 2304 pixels for real-time X-ray beam monitoring with frame rates as fast as 100 Hz. The system provides quantitative beam diagnostics for synchrotron facilities, enabling rapid beam focusing and continuous monitoring of beam position and structure, and may facilitate beam­line automation efforts.

## 1. Introduction

Accurate real-time measurements of X-ray beam position, intensity, and stability are critical for optimizing the performance of existing and next-generation synchrotron sources. Indeed, integrating real-time feedback of X-ray beam properties into control systems for beam­line optical elements and sample positioning is a key objective for next-generation beam­lines as optimization methods using machine learning and AI methods accelerate experiment throughput and reliability (Parkinson *et al.*, 2024 ▸). However, the development of in-beam monitors capable of meeting performance metrics across a wide range of X-ray fluences faces several challenges, including low absorption, high radiation tolerance, wide dynamic range, and sufficient spatial resolution.

This article presents characterization data from a pixelated diamond detector developed to address these needs and enable high-resolution monitoring of beam position, flux, and spatial distribution in real time. This builds on prior work by Zhou *et al.* (2015 ▸), which showed promising results with an early imaging diamond X-ray detector prototype. In this work, we present a commercial video rate imaging in-beam monitor and characterize its linearity over a wide dynamic range, and present case studies on measuring beam drift and imaging in-beam features. This work provides a foundation for future use cases, such as operating real-time beam image acquisition for automated beam control. The data presented in this work were col­lected using the ‘ClearXCam’, a commercially available <!?tlsb=-0.17pt>pixelated beam-positioning monitor product de­signed and man­ufactured by Advent Diamond, Inc. (https://adventdiamond.com/).

In recent years, diamond has been used increasingly as a material for semi-transparent beam­line-positioning monitors because of its unique properties. Its low X-ray absorption and mechanical strength allow for thin diamond detectors (30 to 50 µm used in this work) that provide high transparency, for example, over 90% at 6.3 keV and over 95% at 7.9 keV (Bohon *et al.*, 2010 ▸). Diamond’s wide band gap (5.47 eV), high carrier mobility for electrons and holes, and high thermal conductivity support stable X-ray detection (Berdermann *et al.*, 2010 ▸; Gaukroger *et al.*, 2008 ▸; Bergonzo *et al.*, 2000 ▸; Kagan, 2005 ▸; Keister & Smedley, 2009 ▸). As beam­line operations increasingly incorporate machine learning and automation (Parkinson *et al.*, 2024 ▸), there is growing need for real-time high-resolution beam diagnostics. There are several use cases for the instrument. It was primarily designed as a transmission-type in-beam device that allows beam monitoring and diagnostics during downstream experiments. In addition, it can be used as a removable device during beam tuning, R&D, and beam conditioning. Despite the need for high-resolution sensors, most commercial diamond beam monitors still use four-quadrant designs. In this work, we demonstrate a pixelated diamond detector with enhanced imaging capabilities suitable for real-time beam monitoring at synchrotron facilities.

## 2. ClearXCam design and operation

**Figure 1:**
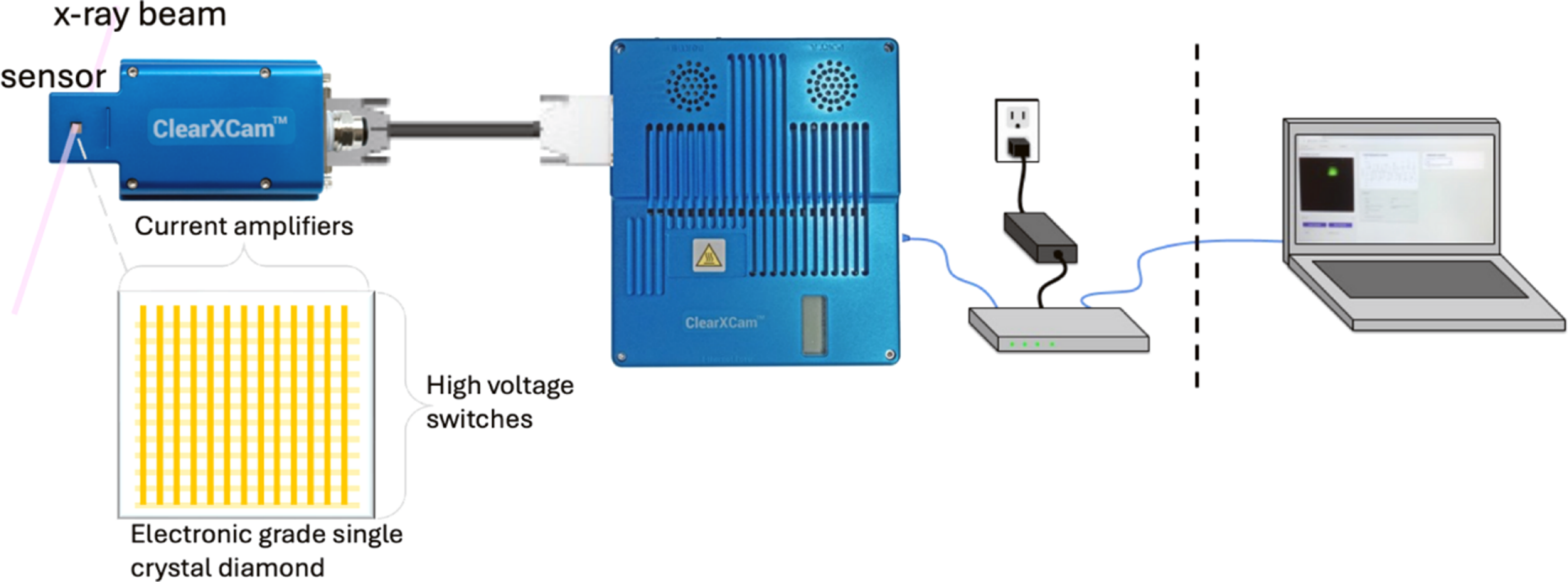
The sensor is an electronic-grade diamond patterned with 48 stripes on both the front and the back sides. Each stripe on one side is connected to an individual current amplifier channel, while the orthogonal stripes on the opposite side are connected to bias switches. These switches are activated sequentially, while current is read continuously, forming a 2D image of the beam. This cycle is repeated to enable video rate image capture. Readout electronics transmit data to a control computer over a network, and the system software includes an API and EPICS support. The sensor is positioned directly in the X-ray beam path, allowing the beam to pass through the diamond while being imaged in real time.

An overview of the experimental setup and equipment is shown in Fig. 1 ▸. The ClearXCam system consists of a diamond sensor featuring 48 metal stripes on the front side and another set of 48 stripes on the back side, oriented orthogonally to the front side stripes. The diamond sensor is housed within a dedicated sensor module, which connects *via* an SCSI cable to the readout electronics. The sensor is positioned in the path of the X-ray beam, so that the beam passes through the sensor. The data can be accessed *via* a graphical user interface, an API, or EPICS for real-time analysis.

### 2.1. Detector design and fabrication

Single-crystal electronic-grade diamond substrate is used as the sensing material, which is metalized with 48 stripes on either side to form a metal–insulator–metal (‘MIM’) detector structure. The sensor design uses 25 µm-wide stripes separated by 25 µm gaps. The nominal active area of these sensors, as defined by the area in which stripes overlap, is 2.373 mm × 2.373 mm.

**Figure 2:**
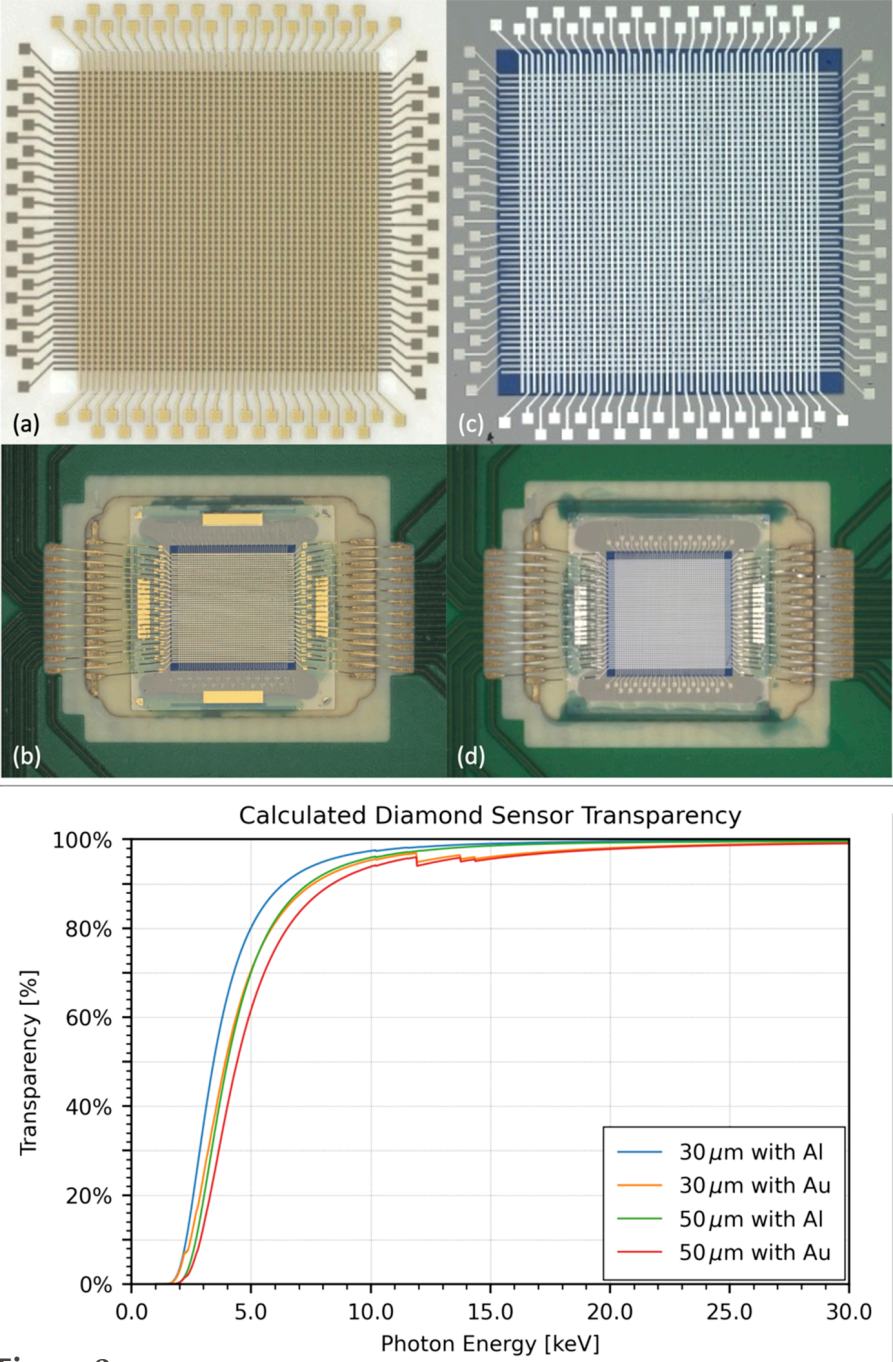
Top: sensor post fabrication and packaging. Microscope images of pixelated diamond sensor showing a 48 × 48 pixel array. (*a*) The Au (gold) electrode metallization on a 4.5 mm × 4.5 mm sCVD substrate; (*b*) the packaged sensor with Au contact metal and wire bonds; (*c*) the Al (aluminium) electrode metallization; (*d*) the packaged sensor with Al contact metal and wire bonds. Bottom: transparency of the sensor calculated for 30 and 50 µm-thick diamond capped with aluminium or gold. These values are calculated from tabulated mass attenuation coefficients (https://henke.lbl.gov/optical_constants/filter2.html). The step visible in the gold traces near ≃12 keV corresponds to the Au L-absorption edges while the aluminium capped option does not show such sharp features. A 30 µm diamond sensor with Al contacts is ≲90% transparent by 6.3 keV and ≲95% by 7.9 keV; the thicker 50 µm/Au configuration reaches these transparencies at roughly 9 and 12 keV, respectively.

Fabrication of the detector begins with a thin (30–50 µm) electronic-grade type IIa substrate with (100) surface orientation and average surface roughness of less than 0.6 nm. Electronic-grade substrates are chosen for their low nitrogen incorporation and defect densities. Following this, the front and back metallization steps are completed *via* a photolithography process, followed by metallization with a refractory metal contact interlayer between the diamond and the capping metal. The capping metals studied in this work are aluminium and gold. To protect the stripes and inter-stripe areas, and promote sensor durability (*e.g.* inhibit diamond surface conduction that can sometimes arise in ozone-rich environments), a proprietary dielectric passivation layer is added. After front-side fabrication is completed, this process is repeated on the backside, with the stripes rotated 90° relative to those on the front side to complete the sensor structure. Microscope images of the gold- and aluminium-capped sensors before and after packaging are shown in Fig. 2 ▸.

**Figure 3:**
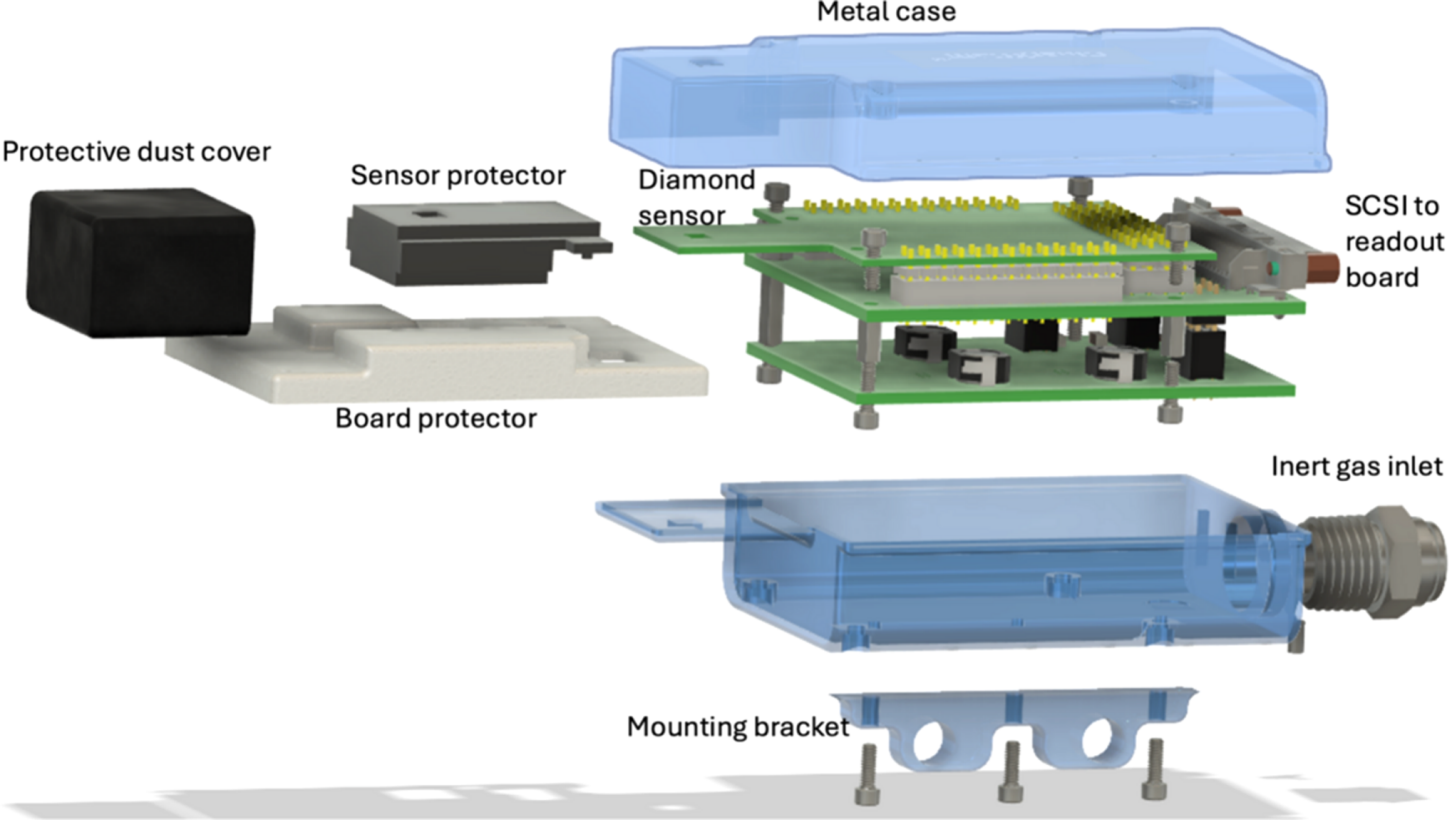
Exploded rendering of the diamond detector module assembly. The diamond sensor is positioned in the ‘Tee’ area on the left. The case includes a gas inlet for optional inert gas flow. The module connects *via* an SCSI cable to the readout electronics.

Once fabricated, the detector is mounted to a custom package (made of a composite ceramic material) using a non-conductive die-attach epoxy. Wire bonds establish the electrical connections between the package and the sensor, as shown in Figs. 2(*b*) ▸ and 2(*d*) ▸. After wire bonding, the packaged sensor is seated onto a secondary board assembly containing bias switches and connections for motherboard readout, all enclosed within a compact metal case with a gas supply inlet for an optional inert gas flow (Fig. 3 ▸).

### 2.2. Readout mechanism

As with any diamond X-ray sensor, photons absorbed in the diamond layer generate electron–hole pairs. An applied electric field drives these charge carriers toward metal contact stripes (ground and DC bias), where the resulting current signal is read by an amplifier circuit. Within the operating bias voltage range used (−10 to −40 V), increasing the bias strengthens the applied electric field across the diamond and generally improves charge collection efficiency by sweeping carriers to the contacts more quickly and reducing charge recombination or trapping.

To generate video rate images, the ‘bias side’ of the diamond detector sets each stripe to either high voltage or ground, controlled by a single-pole double-throw (SPDT) switch. This enables one or more stripes to be active at any given time (single-stripe mode or multi-stripe mode). On the ‘ADC side’, each stripe connects to its own amplifier and analog-to-digital converter, which continuously reads the signal produced through the metal stripes.

The readout electronics automate the imaging process as follows: (i) bias the first stripe and ground all others; (ii) sample the diamond current from all channels to create a 1D beam profile; (iii) ground the biased stripe; (iv) sample all channels again; (v) move to the next stripe and repeat the same process. This sequence continues until all 48 stripes have been sampled, constructing an image frame of 48 × 48 pixels. This sampling cycle repeats continuously at a user-defined frame rate to produce a streaming video (2304 pixels per frame) of the X-ray beam as it interacts with the detector.

Under the default single-stripe settings, the duty cycle is 1/48, since the bias stripes are changing and the amplifiers are always on. In this mode, each stripe is biased only during its turn in the 48-step readout sequence; the reported per-frame ‘total current’ is the frame-summed signal obtained by adding the sequential stripe measurements used to build each frame. We do not apply an additional duty-cycle normalization to the measured total current.

In multi-stripe mode (when the number of active stripes is greater than one), the duty cycle increases and more than one stripe is biased simultaneously (‘Concurrent Stripe’ mode options include 5, 13, or 25 stripes), with the current attributed to the central stripe. Following the same imaging process, all biased stripes shift by one position, and the total current of all stripes is again attributed to the new central stripe. This can improve sensitivity by increasing the biased collection area at each sampling step, thereby collecting charge carriers generated over a larger area before they recombine or become trapped.

In regions of the diamond where no bias is applied (no active stripes), recombination of electron–hole pairs occurs before collection, and thus do not contribute to the sampled current.

### 2.3. Readout electronics

This sensor module connects *via* a commercially available SCSI-style cable to a custom readout electronics system containing the front-end modules (amplifiers and analog-to-digital converters) and a microcontroller. Additionally, the readout module is integrated into a case designed for EM noise rejection and optional airflow cooling, and is powered by a 12 V DC barrel jack (with optional power-over-Ethernet). The unit is preloaded with firmware and has a form factor of 170 mm (W) × 190 mm (L) × 24 mm (D).

The data acquisition system is designed to be highly con­fig­ur­able in real time, with adjustable ADC sample rate, imaging frame rate, switching con­figuration, and other parameters. The ADC is driven by a 40 MHz master clock, and the resulting frame rate is set by 40 MHz divided by the product of the programmed sample period, the number of samples per high-voltage state, the length of the high-voltage sequence, and the number of detector stripes.

## 3. Experiment set up at BNL’s NSLS-II synchrotron

**Figure 4:**
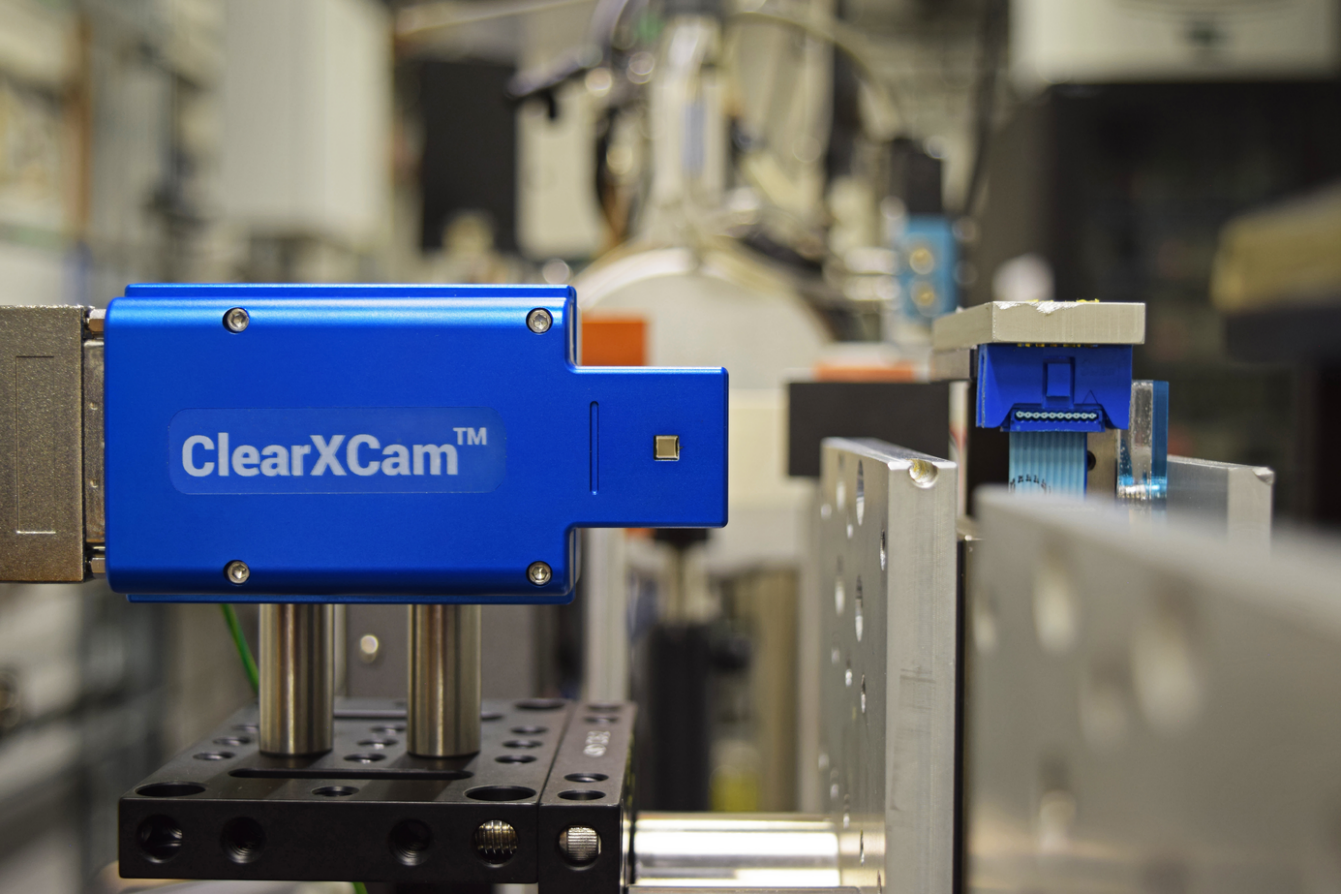
Photograph of the setup at the NSLS-II 17-BM beam­line. The ClearXCam sensor is positioned in the X-ray beam path and is mounted on a motorized stage for alignment and stability. This setup enables in-beam monitoring.

The performance of the unit was assessed at beam­lines 17-BM (XFP) and 4-BM (XFM) at the National Synchrotron Light Source II (NSLS-II) at Brookhaven National Laboratory. The 17-BM beam­line delivers an intense broadband pink beam (4.5–16 keV) from a three-pole wiggler source, with adjustable vertical focusing using a toroidal mirror in the beam­line front end (Asuru *et al.*, 2019 ▸), while 4-BM supplies a microfocused 1–5 µm pink beam (12–20 keV) using Kirkpatrick–Baez mirrors. For detector testing at 17-BM, pinhole apertures (2.0 mm, 1.5 mm, 1.0 mm, 500 µm, 300 µm, and 150 µm) were mounted on a motorized *x–y* translation stage, allowing the use of a range of pinhole sizes. Aluminium filters of various thicknesses were placed upstream of the pinholes to attenuate the incident X-ray beam. The detector itself was positioned furthest downstream, mounted to a second motorized two-axis *x–y* translation stage, with a nitrogen gas purge connected to the sensor module inlet to provide continuous nitrogen flow and prevent ozone buildup near the sensor. A photograph of the set-up at XFP is shown in Fig. 4 ▸.

### 3.1. Beam imaging

**Figure 5:**
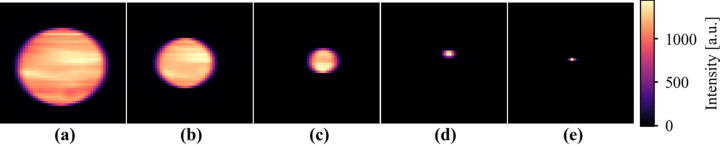
Beam imaging captured by the ClearXCam for beams with varying diameters. (*a*)–(*d*) Images from the XFP beam­line using pinholes of (*a*) 1.50 mm, (*b*) 1.0 mm, (*c*) 500 µm, and (*d*) 150 µm. (*e*) Image from the XFM beam­line with a ∼2 µm beam. Features seen in parts (*a*)–(*c*) are attributed to in-beam structure, likely introduced by either the three-pole wiggler source or upstream beam­line optics.

We first validated that the imaging sensor can resolve beam morphology across a range of beam diameters. Fig. 5 ▸ shows a series of images captured at the XFP and XFM beam­lines. Figs. 5 ▸(*a*)–(*c*) show the measured structure in the XFP beam in the form of striations, which may originate from the beam­line’s three-pole wiggler source properties or the front-end mirror optic. Fig. 5(*e*) ▸, acquired at the XFM beam­line with a beam size of approximately 2 µm, shows that while beam imaging is limited by the effective pixel size of 50 µm × 50 µm, the sensor can still detect beam position for beams smaller than the pixel size. When a beam smaller than the effective pixel falls near the gap between electrodes, charge sharing occurs between the neighboring pixels, and the position of the beam can be approximated from the normalized differences of their signals in a similar method as a four-quadrant diamond beam-position monitor determines position from its electrode currents.

### 3.2. Linearity

**Figure 6:**
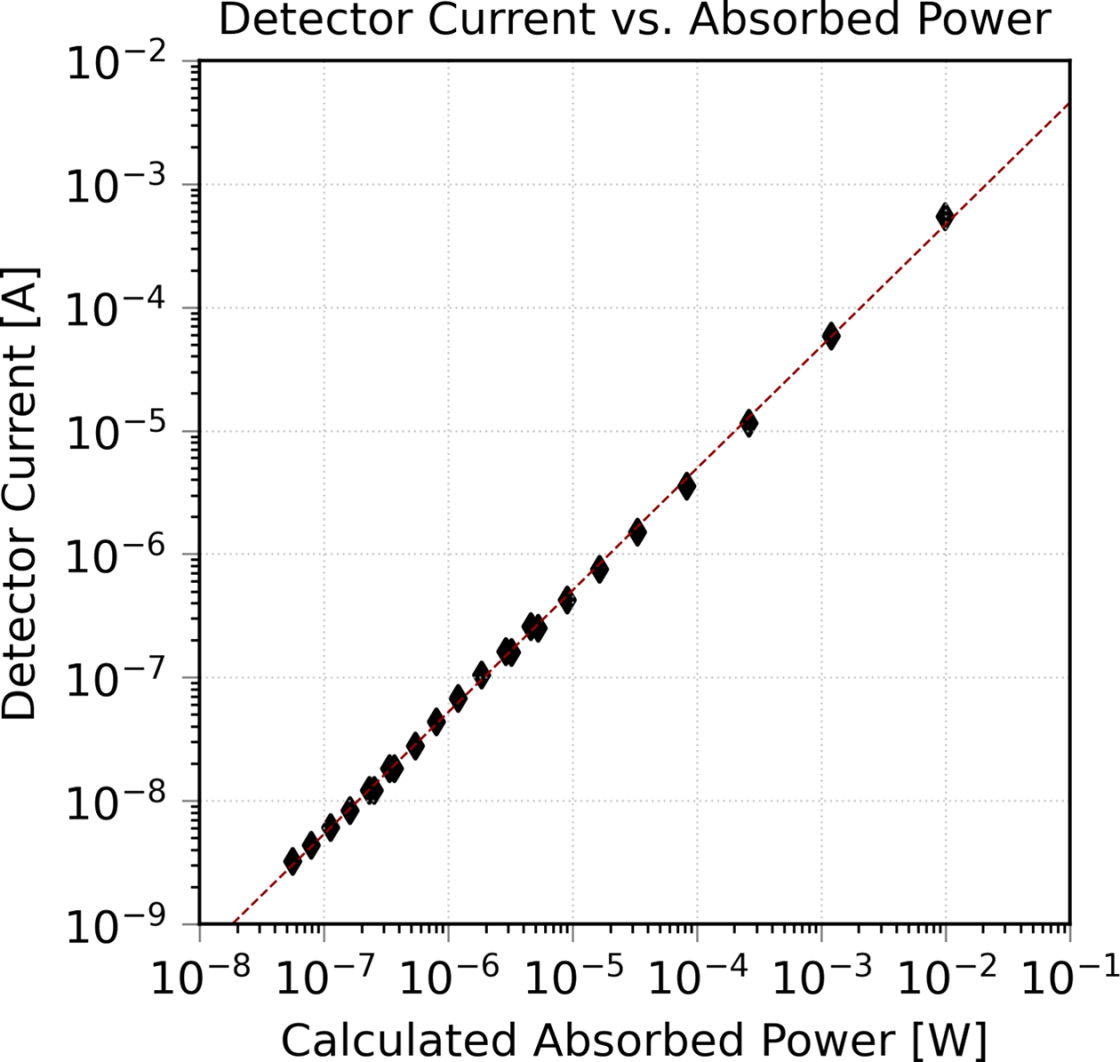
Diamond detector linearity characterization. Log–log plot of total detector current [A] *versus* calculated absorbed power [W] using the ClearXCam imaging monitor using a pink beam at 17-BM with a 1.5 mm pinhole beam spot size. The data were collected in single-stripe collection mode. The fit (red line) yields *I*_1_ = (4.42 × 10^−2^)Φ^0.9878^, where *I*_1_ is the detector current and Φ is the calculated absorbed power, confirming a nearly linear response across five orders of absorbed power. We note that the incident beam has a Gaussian lateral profile, while the absorbed power calculation assumes a uniform distribution. This leads to a slight underestimation of absorbed power and a small rightward shift of the data relative to a unity slope line, though the linearity remains unaffected.

We next demonstrate a linearity experiment using the ClearXCam at the XFP beam­line. To assess the dynamic range of the detector, we varied the beam flux using aluminium attenuators ranging from 0.91 µm to 40.00 mm in thickness. The absorbed power in the sensor was calculated from the known spectral flux distribution of the beam­line, which is provided as discrete energy bins (∼0.1% bandwidth, up to ∼30 keV). For each energy bin, we applied the transmission of the aluminium attenuators and the metal electrode stack of the sensor, computed the resulting absorbed flux in the diamond bulk, and then integrated the absorbed flux × energy over all bins to obtain the total absorbed power. We then plotted the detector current as a function of absorbed power. The data (Fig. 6 ▸) show a highly linear response, with a fit of *I*_1_ = (4.42 × 10^−2^)Φ^0.9878^, where *I*_1_ is the detector current and Φ is the calculated absorbed power.

### 3.3. Durability, operational limits, and vacuum com­pat­i­bility

While we have not yet carried out a systematic lifetime study, the off-the-shelf ClearXCam has operated on synchrotron beam­lines over a wide range of beam intensities, including exposure to focused and unfocused beam without attenuation, corresponding to fluxes up to ∼10^16^ photons s^−1^ for up to 60 s. In terms of signal dynamics, a temporary and localized afterglow may be observed after higher intensity beam exposure likely due to trapped charge at defect sites in the diamond. After the sensor is allowed to rest (typically overnight), the response returns to baseline, and we have not observed long-term performance degradation following high-intensity exposure or continuous use. A more extensive lifetime study under the most extreme beam conditions remains future work.

The present commercial ClearXCam package is not designed for UHV operation. In-vacuum or front-end use in UHV and/or inert-gas environments is in principle feasible but would require additional engineering, including UHV-com­pat­i­ble packaging, cooling, and signal feedthroughs. With adequate cooling, we expect the diamond itself to remain operational even in very intense beams, while the metal contacts are likely to be the limiting component. With targeted design modifications, such as employing conducting stripes made from metallic or doped diamond, a nearly all-diamond sensor could be realized for operation in the most demanding front-end environments, with signals routed outside the vacuum vessel *via* appropriate connectors. Potential users interested in UHV com­pat­i­ble configurations are encouraged to contact Advent Diamond directly.

## 4. In-beam case studies

In this section, we present a series of in-beam case studies demonstrating the ClearXCam’s performance under varying beam conditions and operational modes.

### 4.1. Rapid beam focusing

**Figure 7:**
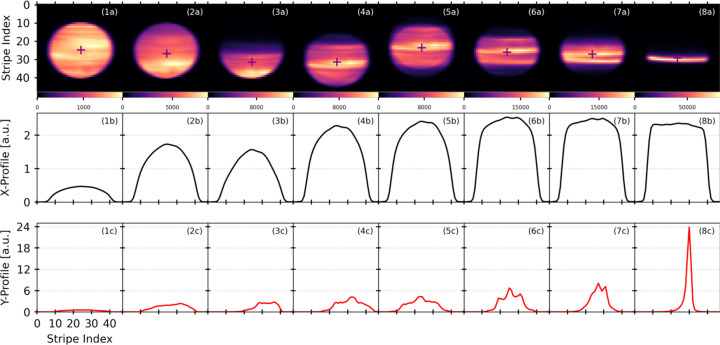
Top (1*a*)–(8*a*): film strip sequence showing ClearXCam images recorded during a vertical focusing event at beam­line 17-BM, spanning approximately one minute as the front-end toroidal mirror was adjusted. This sequence of images shows how the beam evolves during focusing. Bottom rows (*b*) and (*c*): beam profiles in the horizontal (*x*) and vertical (*y*) directions, shown as cross-sections of beam intensity (slices through the 2D beam image) taken at the center of mass (COM), demonstrate a narrowing of the vertical peak width as the beam is brought into focus (the XFP beam does not focus in the horizontal direction). The crosshair is placed at the COM of the beam. The reduction in observed intensity between horizontal profiles (2*b*) and (3*b*) is attributed to motion of the beam on the detector during focusing as the mirror is adjusted. This case study illustrates the potential of pixelated diamond detectors for in-beam diagnostics and their com­pat­i­bility with automated beam-tuning optimization workflows.

To illustrate the capabilities of in-beam imaging, a focusing experiment was performed at beam­line 17-BM using the ClearXCam system, while the front-end mirror was adjusted to bring the beam into vertical focus. Beam images were continuously recorded during the focusing sequence and are shown in Fig. 7 ▸ (top). As shown in Fig. 7 ▸ (middle and bottom), the data can also be plotted in real time to display the horizontal (*x* direction) and vertical (*y* direction) beam profiles. As expected, the vertical width of the beam narrows as it comes into focus. In this case, vertical focus was achieved in under one minute through manual mirror adjustments. Even faster focusing could be achieved with automated feedback enabled by the instrument. These results highlight the potential of pixelated diamond detectors for in-beam diagnostics and point to future integration with optical control systems for automated beam tuning.

### 4.2. Case study: fast imaging mode

**Figure 8:**
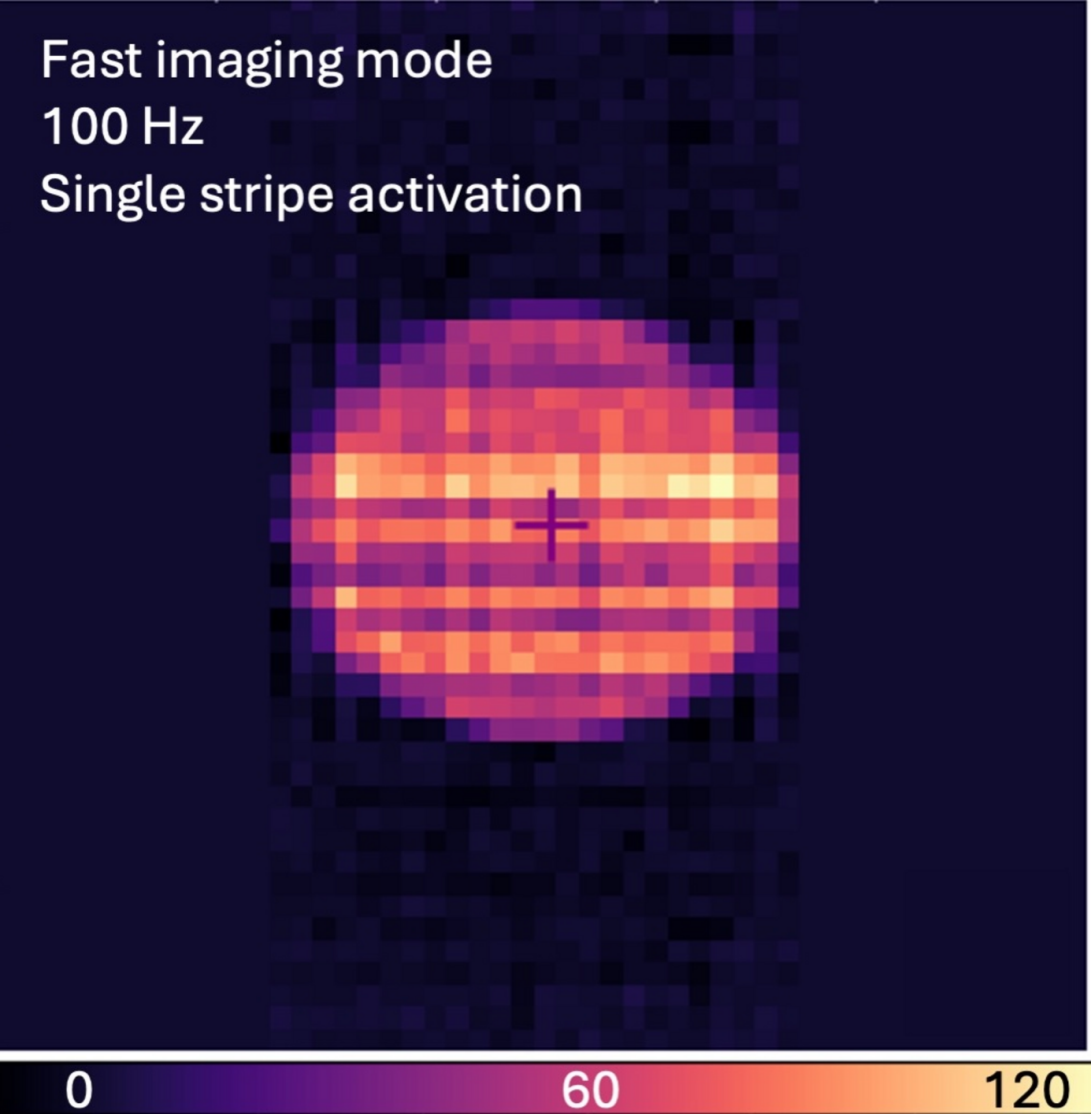
Frame from imaging at 100 Hz in single-stripe mode at the XFP beam­line, using a 1.0 mm pinhole and a 2.4 mm aluminium attenuator to reduce flux to ∼3 × 10^13^ photons s^−1^. Some X-ray beam features such as striations remain visible, though the tradeoff to sampling at higher frame rates is worse signal-to-noise, as expected.

Some beam­line imaging use cases, such as automating alignment tasks, require fast imaging. To evaluate this ca­pa­bil­i­ty, we exposed the sensor to the XFP beam­line, using a 1.0 mm diameter pinhole to define the beam spot size and attenuated the beam with a 2.4 mm thick aluminium plate. The capture rate was set to 100 Hz in single-stripe mode. One frame of the imaging data from this experiment is shown in Fig. 8 ▸. Some beam features, such as striations, remain dis­cernible at this imaging rate though the signal-to-noise is affected as expected. The available video capture rate of the instrument ranges from 0.5 to 100 Hz.

### 4.3. Case study: positioning resolution applied to characterizing beam drift

**Figure 9:**
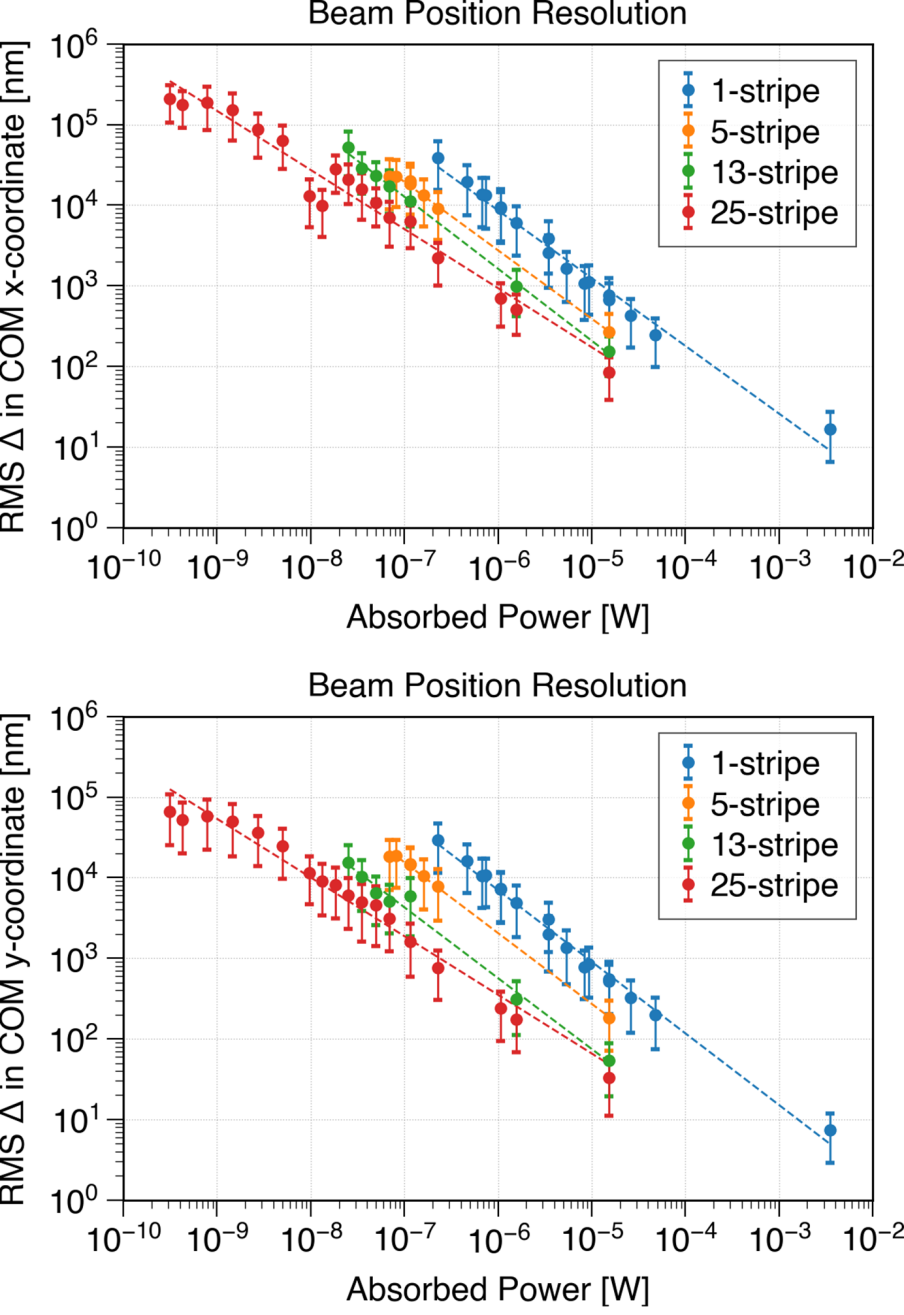
Top (bottom): change in the *x* coordinate (*y* coordinate) of the center of mass (COM) of the beam *versus* absorbed power in multi-stripe mode with 1 (blue), 5 (orange), 13 (green), and 25 (red) simultaneously biased stripes. The data were collected with no intentional movement between the beam and the sensor. The data show that beam drift sensitivity improves with increasing absorbed power. Power-law fits (dashed lines) are shown for each mode. At an absorbed power of ∼2.0 × 10^−5^ W (beam flux ∼4.0 × 10^1^^1^ photons s^−1^) in 1-stripe mode, the ClearXCam can resolve ∼1 µm vertical beam drift, while in 25-stripe mode, similar precision is achieved at ∼8.5 × 10^−6^ W. At an absorbed power of ∼8.0 × 10^−4^ W (beam flux of ∼2.0 × 10^13^ photons s^−1^) in 25-stripe mode, the ClearXCam resolved ≲100 nm, which is less than 0.01% of the 1.5 mm beam diameter. The lower values for drift in the *y* direction are expected to be real and not an artifact of the instrument, showing a use case for in-beam diagnostics. We note that, given the transparency of the sensor, the absorbed power is about ∼1% of the beam incident power.

As an additional case study demonstrating the ClearXCam’s capabilities, we evaluated its sensitivity to beam drift by analyzing the root-mean-square (RMS) change in the center of mass (COM) of the beam in both single-stripe and multi-stripe operating modes. For this measurement, the sensor and upstream optics were kept fixed so that any variation in beam COM between frames reflects a combination of intrinsic beam motion and residual noise from the ClearXCam readout electronics. For each aluminium attenuation configuration (corresponding to a given absorbed power on the horizontal axis of Fig. 9 ▸), we acquired continuous time series data over ∼2 min intervals at the default frame rate and computed the COM for every frame. We then formed the frame-to-frame COM differences and took the RMS of those differences as a measure of beam-position stability. Configurations with 1, 5, 13, and 25 active stripes are shown in blue, orange, green, and red, respectively, in Fig. 9 ▸. Using multi-stripe mode, the system measured beam position across a dynamic range spanning five orders of magnitude. For example, at an absorbed power of approximately 8 × 10^−6^ W (beam flux ∼2 × 10^13^ photons s^−1^) and with 25 stripes activated, the measured RMS change in the COM was about 100 nm, which is less than 0.01% of the 1.5 mm beam diameter. Another outcome of the case study is observing that for this beam, more position variation is observed in the *x* than in the *y* direction, indicating an asymmetry in the beam-position stability. We note that given the transparency of the sensor, the absorbed power is approximately 1% of the incident beam power.

## 5. Conclusions

As synchrotron facilities continue to upgrade with increased automation and higher brightness sources, real-time high-resolution beam diagnostics become increasingly necessary. In this work, we evaluated the newly commercialized ClearXCam’s performance in monitoring beam position, in-beam structures, and intensity. The results demonstrate its suitability for beam­line environments that require continuous feedback of beam parameters to support experimental control and accelerate tasks such as beam focusing. We also identified parameters to optimize detector performance for different use cases. Higher photon intensities and applications that do not require fine spatial resolution can benefit from the fast acquisition mode, with imaging rates up to 100 Hz. We demonstrated linearity with absorbed power over nearly five orders of magnitude. Additionally, we demonstrated real-time sub-micron beam-drift measurements as another practical application. With EPICS com­pat­i­bility, future work on the ClearXCam may include demonstrating beam­line integration for automated control, complementing autonomous alignment frameworks, such as that recently described by Morris *et al.* (2024 ▸), which require precise real-time beam diagnostics to enable fully automated beam­line tuning.

## Data Availability

The data supporting the findings of this study are available within the article. Additional datasets or raw measurement files used during the current study are available from the corresponding author upon reasonable request.
